# A Case of a 5-Year-Old Boy with a Blauth Type IIIB Hypoplastic Thumb Reconstructed with a Nonvascularized, Hemilongitudinal Metatarsal Transfer

**DOI:** 10.1155/2018/8205285

**Published:** 2018-12-06

**Authors:** Mika Nakada, Kaoru Tada, Tadahiro Nakajima, Masashi Matsuta, Hiroyuki Tsuchiya

**Affiliations:** Department of Orthopaedic Surgery, Graduate School of Medical Sciences, Kanazawa University, 13-1, Takara-machi, Kanazawa 920-8641, Japan

## Abstract

The treatment methods used for Blauth type IIIB hypoplastic thumbs are controversial. We performed a nonvascularized, hemilongitudinal metatarsal bone transfer on a 5-year-old boy with a type IIIB hypoplastic thumb. Despite the child's age, the growth of the thumb was confirmed and the thumb had stabilized. Moreover, growth disorder of the donor toe was not observed. This method is relatively easy to perform. And donor toe deformation can be prevented, because of the preservation of more than half of the metatarsal bone. In our case, the patient was 5 years of age; nevertheless, the epiphyseal line was opened and the grafted metatarsal bone grew. This method is useful in terms of its simplicity and prevention of postoperative complications.

## 1. Introduction

A hypoplastic thumb is a type of radial ray deficiency and is relatively rare. The modified Blauth classification is commonly used to classify the condition, which is based on the atrophy of the thenar muscles, the contracture of the adductor pollicis, and the degree of dysplasia of the metacarpus in the thumb [1]. Type I thumbs have minimal shortening and narrowing. Type II thumbs have thumb-index web space narrowing, hypoplasia intrinsic thenar muscles, and metacarpal joint instability. Type IIIA thumbs have type II features plus extrinsic tendon abnormalities and hypoplastic metacarpal with a stable carpometacarpal joint. Type IIIB thumbs have type IIIA features plus partial metacarpal aplasia with an unstable carpometacarpal joint. Type IV thumbs are floating thumbs, and type V thumbs are absent thumbs [1, 2]. Chow et al. [3] reported a method with the use of nonvascularized, hemilongitudinal metatarsal transfer for Blauth types IIIB and IV. Herein, we report the case of a 5-year-old boy with a type IIIB hypoplastic thumb. Despite the child's age, we successfully performed a nonvascularized hemilongitudinal metatarsal bone transfer based on Chow's method.

## 2. Case Presentation

**Figure 1:**
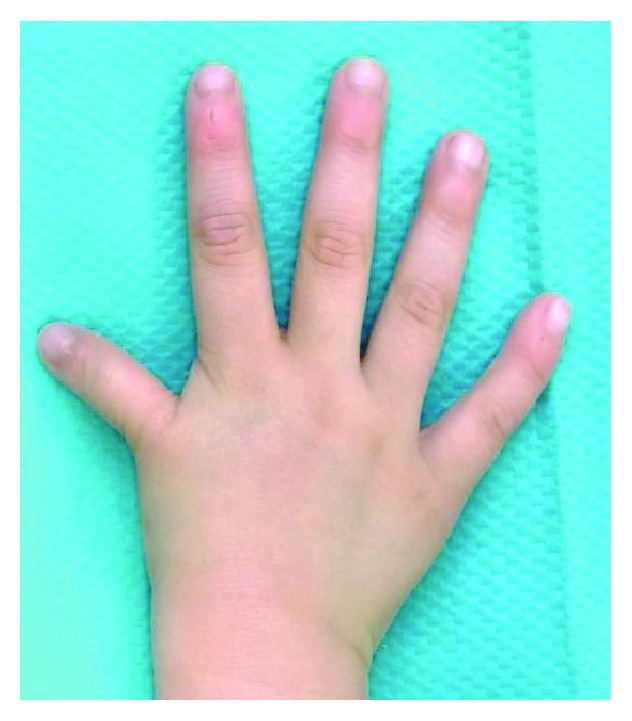
A 5-year-old boy with a hypoplastic thumb.

**Figure 2:**
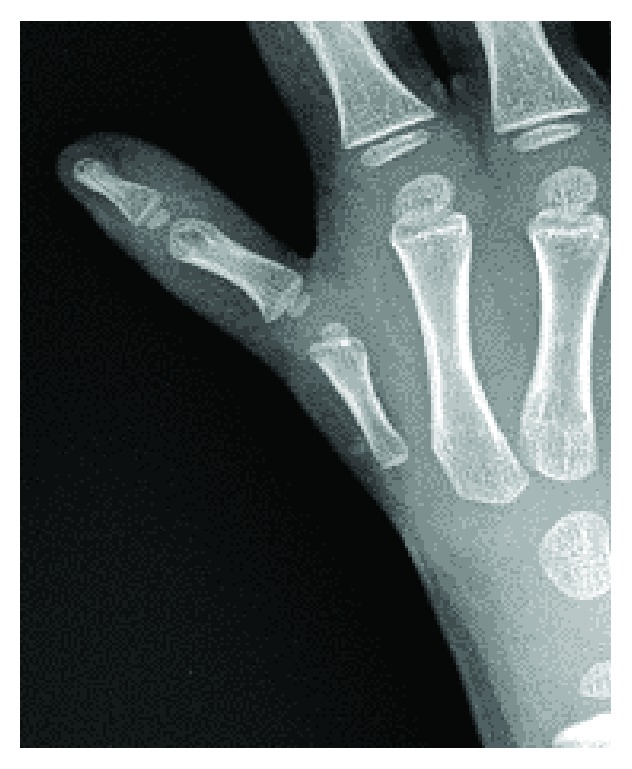
Radiographs demonstrating the type IIIB hypoplastic thumb.

A 5-year-old boy was consulted at the age of 7 months for hypoplasia of his right thumb and active movement disorder. He had no particularly relevant medical history. Active movement of the right thumb was impossible, and X-ray images confirmed his hypoplastic thumb. Since his parents declined surgery, we performed only follow-up observations until he was 5 years old. At this time, his right hypoplastic thumb was more prominent (Figure 1). Active flexion and extension of the interphalangeal (IP) and metacarpophalangeal (MP) joint of the right thumb were impossible, and the carpometacarpal (CMC) joint was unstable. X-ray images revealed hypoplasia of the metacarpal bone and the defective CMC joint of the right thumb (Figure 2). Therefore, the boy was diagnosed as having Blauth type IIIB and performed a nonvascularized, hemilongitudinal metatarsal transfer.

**Figure 3:**
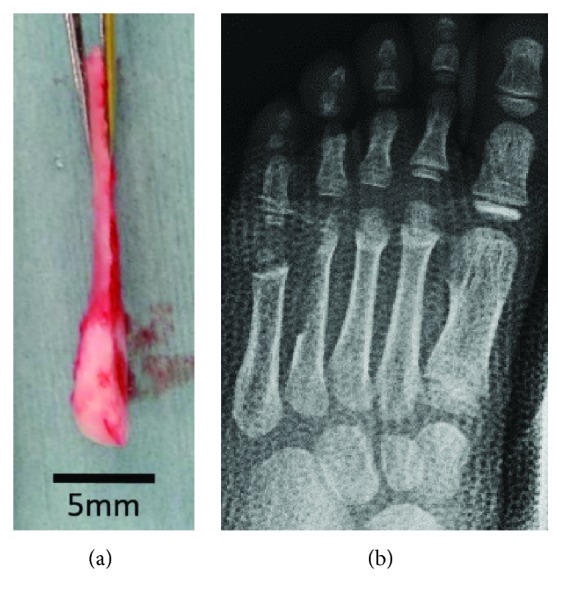
The metatarsal bone cut hemilongitudinally: (a) the cut metatarsal bone and (b) radiograph after the removal of the fourth metatarsal bone.

**Figure 4:**
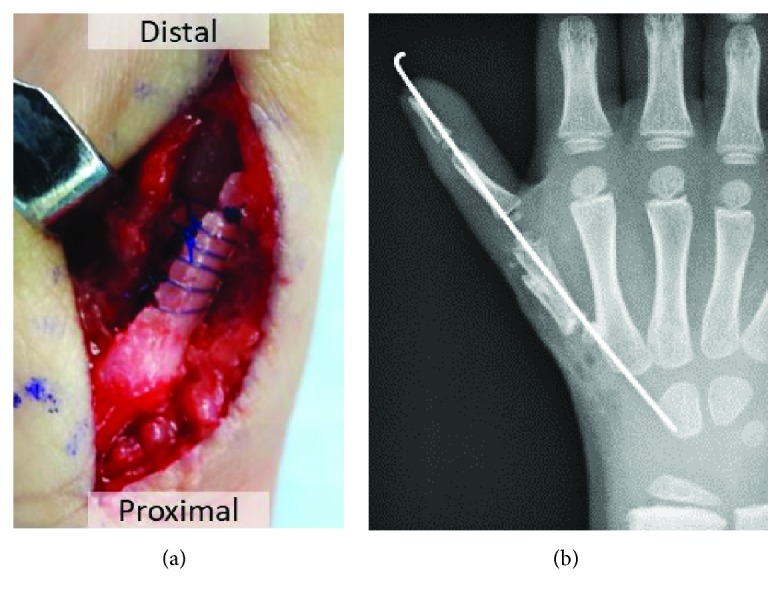
The metatarsal bone transplanted into the metacarpal bone. (a) The metacarpal bone and the cut metatarsal bone were sutured. (b) These were fixed with a Kirschner wire.

**Figure 5:**
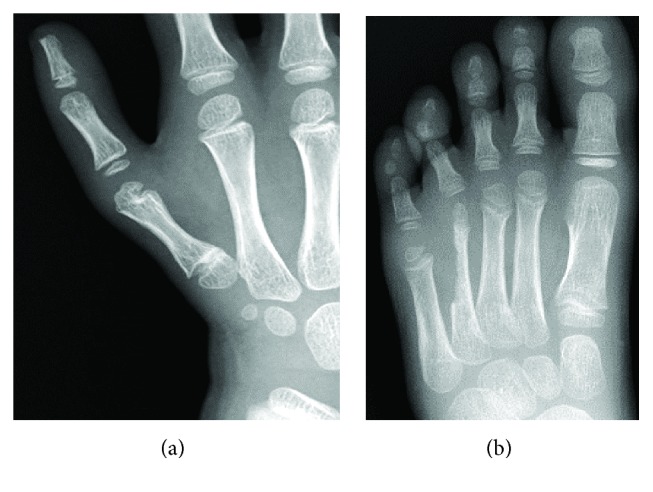
Radiographs of 2 years of follow-up: (a) hand and (b) foot.

**Figure 6:**
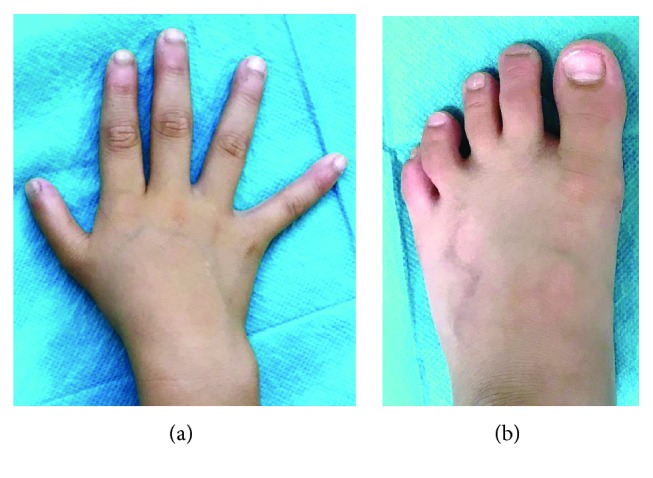
Two years after surgery: (a) hand and (b) foot.

**Figure 7:**
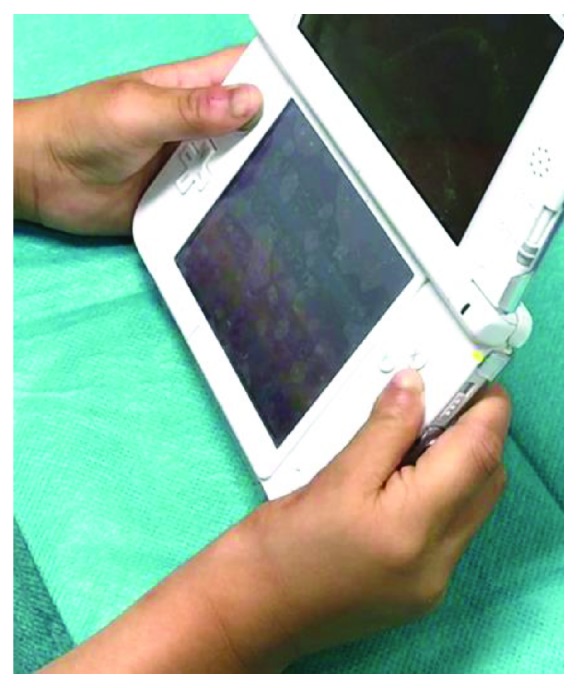
He can push the buttons on a portable game machine.

During surgery, the left fourth metatarsal bone was cut hemilongitudinally from the head to the shaft and the lateral part was removed (Figures 3(a) and 3(b)). The cut metatarsal bone was turned over and transplanted into the metacarpal bone of the right thumb. These were sutured with 5–0 polydioxanone sutures (PDS®, Ethicon) (Figure 4(a)) and fixed with a 0.8 mm Kirschner wire from the distal site (Figure 4(b)). After surgery, he wore a thumb spica brace for 2 months. Immediately after surgery, the metacarpal bone was approximately 20 mm in length, including the transplanted bone. The epiphyseal line was observed, and the metacarpal bone grew to approximately 28 mm in length within 2 years of surgery (Figure 5(a)). The growth of the right thumb was confirmed via gross observation (Figure 6(a)), and the right thumb had stabilized, thereby enabling pinch and opposition. He can hold a pen and push the buttons on a portable game machine. (Figure 7). Moreover, readily notable growth disorder of the donor toe was not observed (Figures 5(b) and 6(b)). Since his parents were satisfied with the thumb's functionality, opponensplasty was not performed.

## 3. Discussion

The clinical presentation of a hypoplastic thumb varies from moderate hypoplasia to the loss of the thumb. As a common treatment option, type I follow-up is performed. Meanwhile, for types II and IIIA, opponensplasty is performed, such as the Huber opposition or flexor digitorum superficialis tendon transfers [4, 5]. Finally, for types IV and V, pollicization is performed [4, 6, 7]. Treatment for these types has generally been well-received; however, the treatment methods used for type IIIB hypoplastic thumbs are controversial [8] and reflect the desire of parents to preserve the thumb [7]. A stable and functional thumb can be created by pollicization. However, it is disadvantaged by the reduction in the number of fingers to four. Meanwhile, additional treatment methodologies (e.g., bone grafting) must be utilized in addition to opponensplasty to establish a stable thumb. In order to establish a stable thumb, bone grafting with the use of a metatarsal bone, toe joint, or fibula is necessary [4]. Furthermore, two-term opponensplasty is required. However, it is difficult to perform vascularized bone grafting, particularly in children. In addition, large bone defects occur after surgery and so growth disorders and toe deformities have been reported as complications of toe joint transplantation and general metatarsal bone grafting [4, 8].

To address these shortcomings, Chow et al. [3] developed a nonvascularized, hemilongitudinal metatarsal bone to metacarpal bone transfer that included the cartilages. She performed nonvascularized, hemilongitudinal metatarsal bone transfer for six hands for patients with an average age of 17.d5 months (11–22 months). She then performed a follow-up for an average of 87.7 months. Chow reported that all grafted bones had grown in length by an average of 1.5 mm per year that grip strength and key pinch ability were 61% and 38%, respectively, of that of the healthy side. Notably, donor metatarsal bone fractures can occur but are healed with casting. Moreover, no growth disorder was observed.

Some studies have reported the application of nonvascularized metatarsal bone grafting. Among them, Goldberg and Watson [9] reported that, following nonvascularized toe to finger transfer that included the epiphyseal plate for congenital finger deficit that 91%, 67%, and 50% of epiphyses were open among patients of 0.5–1.5, 1.5–5, and at least 5 years of age, respectively, at the time of operation, which was based on a mean follow-up of 3.4 years. Meanwhile, Back-Gramcko [10] analyzed the outcomes by the age of a patient at the time of operation. In comparing patients who were younger than 18 months, 18 months to 4 years, or older than 4 years, the study demonstrated that a transplanted metatarsal bone grew the most in the patients aged 18 months to 4 years. Finally, Unglaub et al. [11] reported that the graft bone was mostly resorbed in patients who were at least 4 years of age at the time of operation.

In our case, the patient was 5 years of age and older than the patients who were described in Chow's report. Nevertheless, the grafted metatarsal bone survived and the epiphyseal line was opened. It is considered that the compatibility of the grafted metatarsal bone size and the metacarpal bone was advantageous. Additionally, without performing two-term opponensplasty, we were able to obtain a functional thumb that was satisfactory to the parents. As the limitation, it may cause fractures or deformity of the donor's toe and it is not more functional than pollicization. Overall, this method has two advantages. First, it is relatively easy to perform surgery for nonvascularized bone grafting and no vascular anastomosis occurred. Second, donor toe deformation can be prevented using a hemimetatarsal bone graft, because of the preservation of more than half of the metatarsal bone. As a result, a stable thumb was obtained without deforming the donor toe. This method is useful in terms of its simplicity and prevention of postoperative complications.

## 4. Conclusions

We successfully performed a nonvascularized, hemilongitudinal metatarsal bone transfer on a 5-year-old boy with a type IIIB hypoplastic thumb. Chow's method is a useful method to solve common problems.
